# Supplementary material for: Identification of a DEAD-box RNA Helicase BnRH6 Reveals Its Involvement in Salt Stress Response in Rapeseed (Brassica napus)
Source: Int J Mol Sci. 2022 Dec 20;24(1):2. doi: 10.3390/ijms24010002 (PMC9819673; doi:10.3390/ijms24010002)
Supplement: Supplementary file 1 [file ijms-24-00002-s001.zip › Supplementary Figures.pdf]

Supplementary Figures

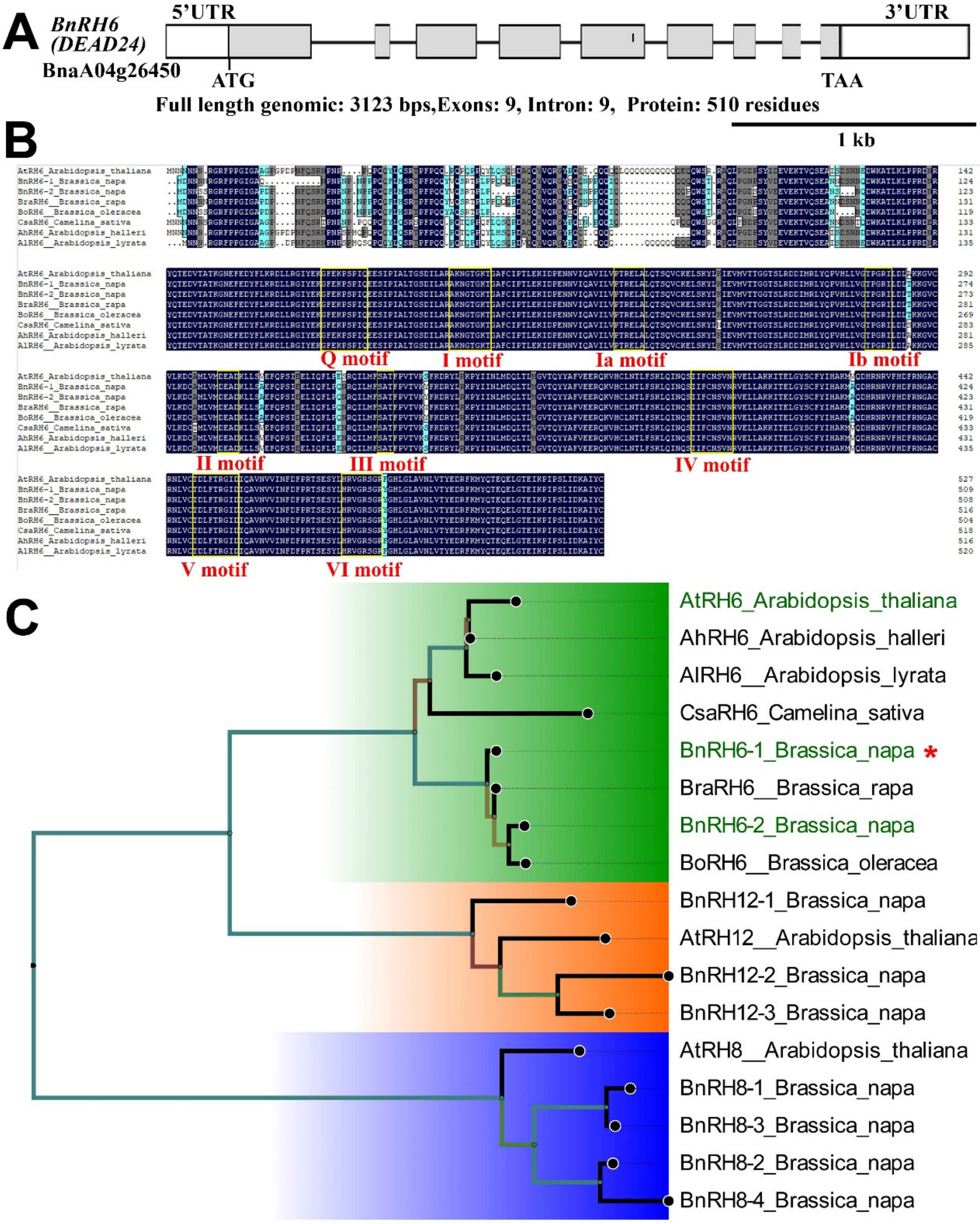

Supplementary Figure S1. Basic information of *Brassica BnRH6* genes. (A): Genomic organization of *BnRH6* the gray rectangle represents exons and line represents introns. The empty rectangle represents untranslated regions (UTR). (B): Comparison of the derived amino acid sequences of BnRH6 with other six species colored by DNAMAN software, nine

conserved domains and potential protein binding sites are marked with yellow line. (C): Phylogenetic analysis of BnRH6 in different species. Proteins from other plant species included DEAD-box ATP-dependent helicase 6 (*Arabidopsis thaliana*), DEAD-box ATP-dependent helicase 6 (*Arabidopsis halleri*), DEAD-box ATP-dependent helicase 6-like (*Camelina sativa*), fgenesh2_kg.4__2779__AT2G45810.1 (AlRH6) (*Arabidopsis lyrata*), DEAD-box ATP-dependent RNA helicase 6 (*Camelina sativa*), DEAD-box ATP-dependent helicase 6-1 (*Brassica napus*), DEAD-box ATP-dependent helicase 6-2 (*Brassica napus*), DEAD-box ATP-dependent helicase 6 (*Brassica rapa*), DEAD-box ATP-dependent helicase 6 (*Brassica oleracea*), DEAD-box ATP-dependent helicase 8 (*Arabidopsis thaliana*), DEAD-box ATP-dependent helicase 8-1 (*Brassica napus*), DEAD-box ATP-dependent helicase 8-2 (*Brassica napus*), DEAD-box ATP-dependent helicase 8-3 (*Brassica napus*), DEAD-box ATP-dependent helicase 8-4 (*Brassica napus*), DEAD-box ATP-dependent helicase 12 (*Arabidopsis thaliana*), DEAD-box ATP-dependent helicase 12-1 (*Brassica napus*), DEAD-box ATP-dependent helicase 12-2 (*Brassica napus*), DEAD-box ATP-dependent helicase 12-3 (*Brassica napus*), the phylogenetic tree was constructed using MEGA6.0. (*, $P < 0.05$, Student's t-test).

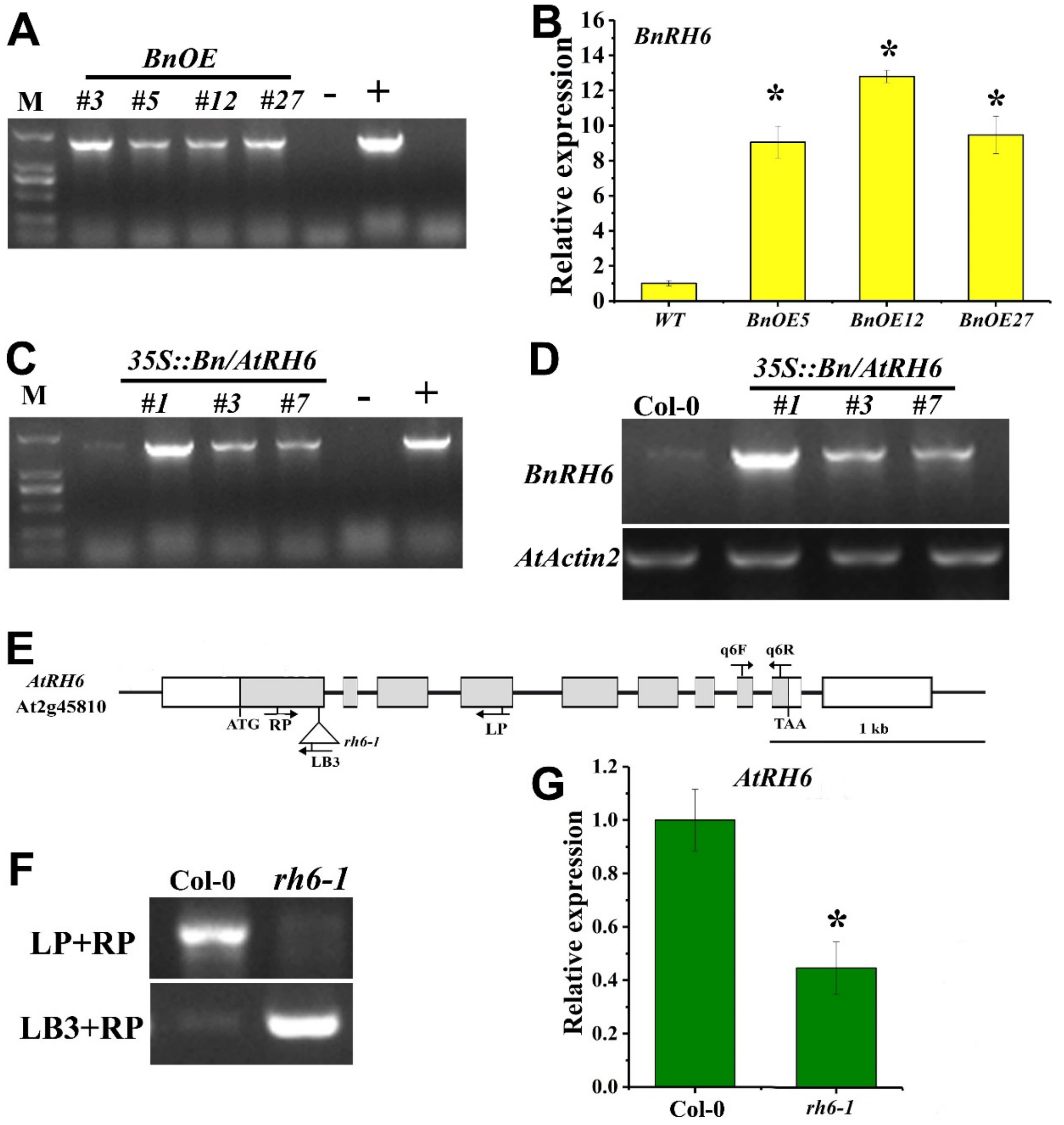

Supplementary Figure S2. Homozygous identification and expression levels of *Brassica* (*BnOE*) and *Arabidopsis (AtOE*) overexpressing *BnRH6* lines, and *Arabidopsis* mutant *rh6-1*. (A): Identification of T3 generation *BnRH6* overexpressing *Brassica* transgenic plants at the DNA level, DNA from 4 transgenic lines (#3, #5, #12 and #27) and WT (-) were used for diagnostic PCR. Plasmid *pCAMBIA 1300-BnRH6* for the positive control (+). (B): qRT-PCR analysis of the *BnRH6* transcripts in wild-type, #5, #12 and #27 lines. (C): Identification of *35S::Bn/AtRH6* transgenic plants at DNA levels. DNA from 3 transgenic lines (#1, #3 and #7) and WT (-) were used for diagnostic PCR. Plasmid *pCAMBIA 1300-BnRH6* for the positive control (+). (D): RT-PCR analysis of the *BnRH6* transcripts in wild-type, 35S::Bn/AtRH6 #1, #3 and #7 lines. *AtActin2* and *BnActin2* were used as internal standards. (E) Schematic diagrams of AtRH6 structure and T-DNA diagnostic PCR and qRT-PCR. The gray rectangle represents

exons and line represents introns. The empty rectangle represents untranslated regions (UTR). Primers used in the genotyping and RT-PCR are shown with arrows. (F) Genotyping PCR for the five mutant lines of *AtRH6* DNA from the insertion lines of *rh6-1*. (G) qRT-PCR analysis of the *AtRH6* transcripts in wild-type and *rh6-1* mutant lines. Vertical bars represent mean values ± SD (*, $P < 0.05$, Student's *t*-test).

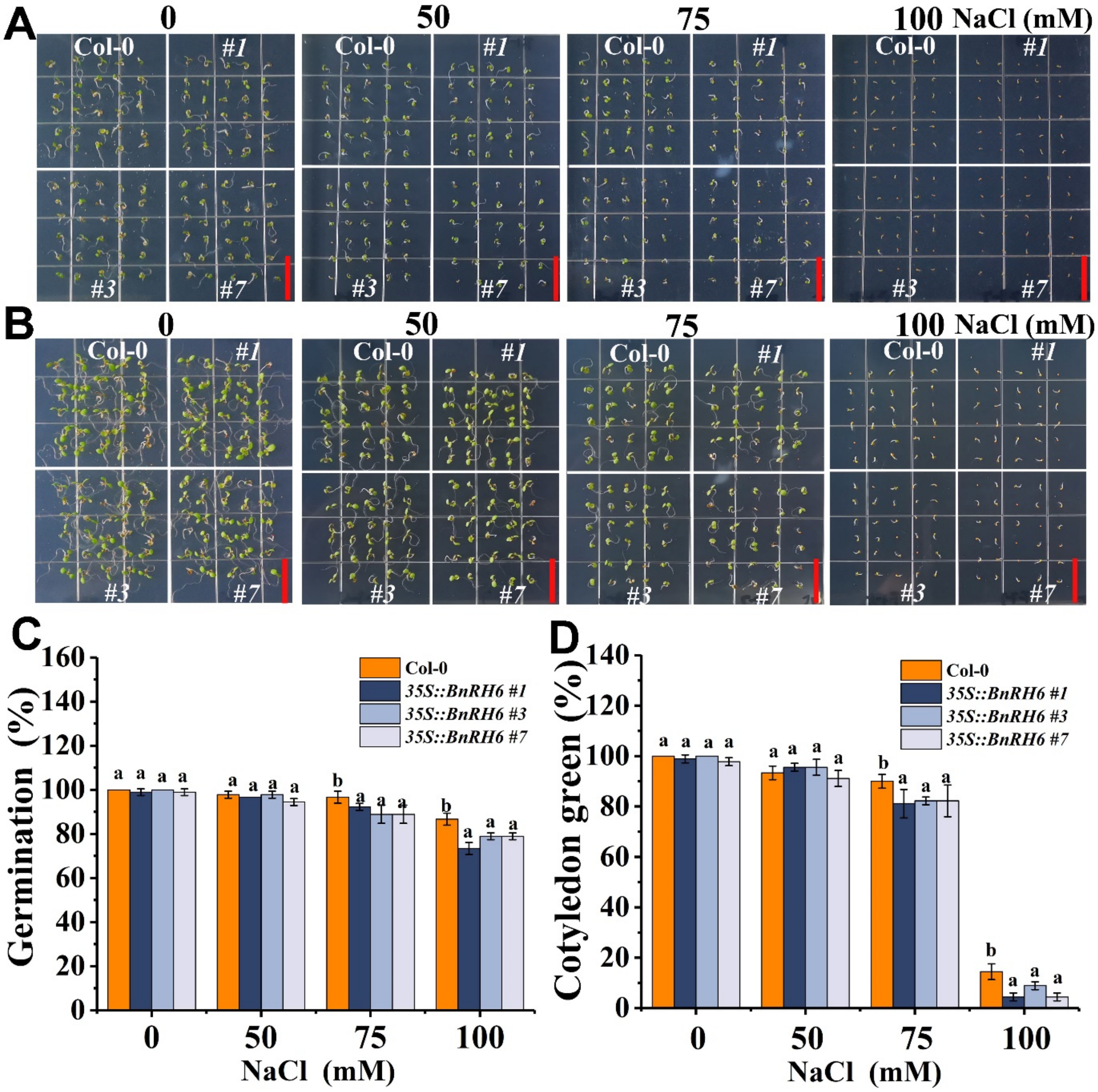

Supplementary Figure S3. Seed germination and cotyledon greening of *Arabidopsis* overexpressing *35S::BnRH6* (*AtOE*s) under salt stress. (A, B): Seed germination and cotyledon greening of *35S::BnRH6* lines. (C): Greening and Cotyledon greening rates of *35S::BnRH6* lines. WT and *35S::BnRH6* seeds at 0, 50, 75, 100 mM NaCl 1/2 MS medium for germination for 4 d. Seven days later, the cotyledon greening was measured. Vertical bars represent mean

values ± SD. Different letters indicate significant differences between WT and AtOE plants (P < 0.05). Scale bar=1.5 cm.

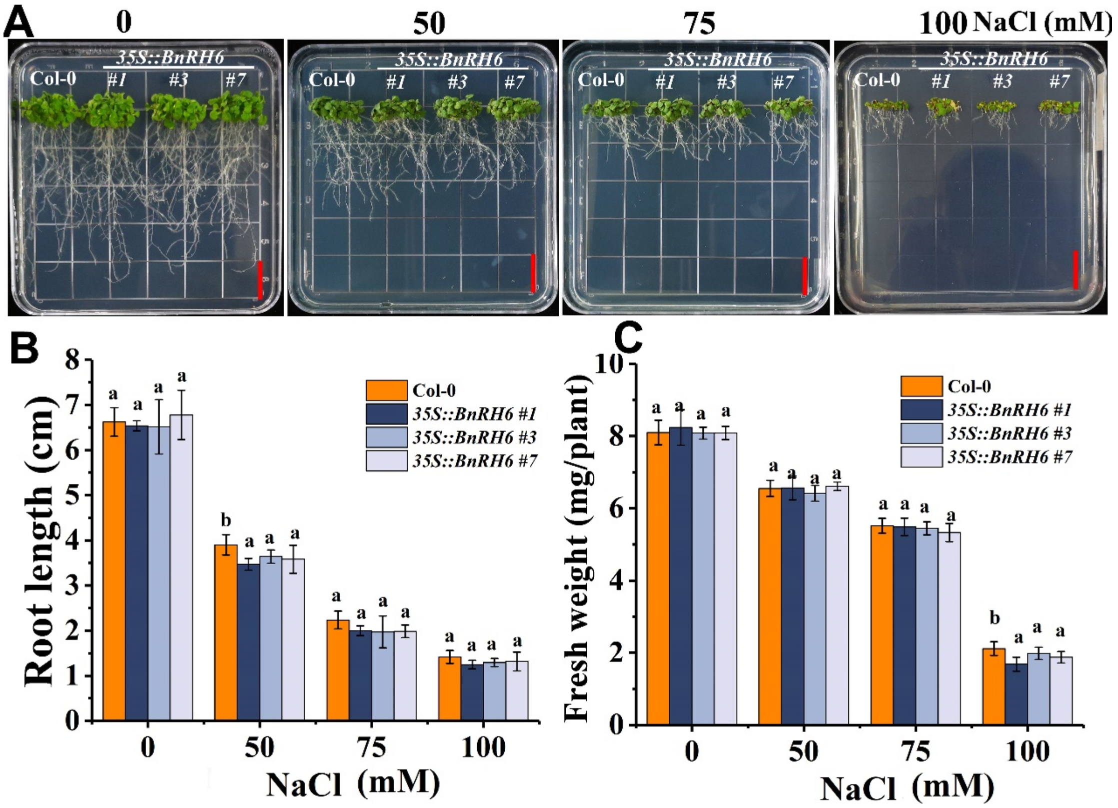

Supplementary Figure S4. Elongation of primary root and plant growth of transgenic *Arabidopsis* overexpressing *35S::BnRH6* (*AtOE*s) under salt stress. (A): Phenotypes of *35S::BnRH6* lines. (B, C): Root length and fresh weight. WT and *35S::BnRH6* seeds were grown in 0. 50, 75, and 100 mM NaCl 1/2 MS medium. 12 days later, the root length and fresh weight were measured. Vertical bars represent mean values ± SD. Different letters indicate significant differences between WT and AtOE plants (P < 0.05). Scale bar=1.5 cm.

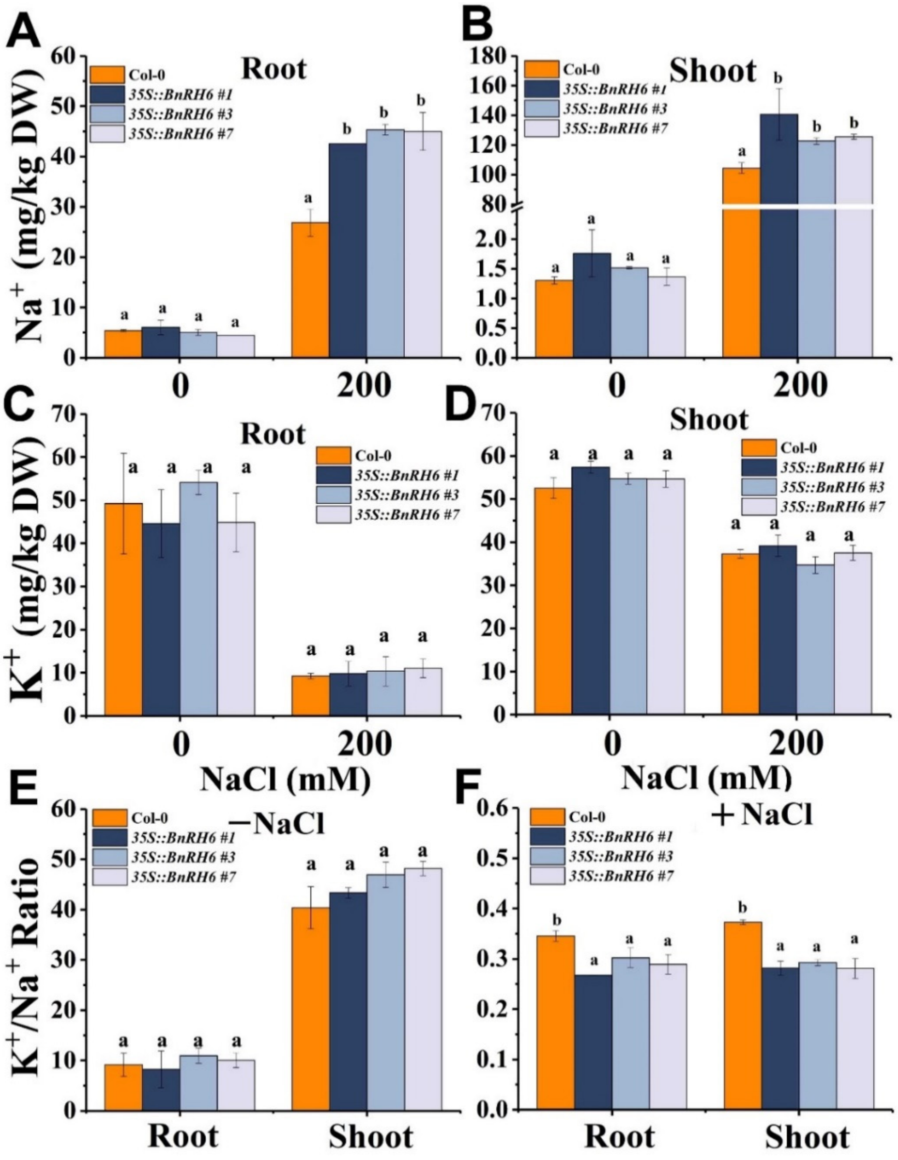

Supplementary Figure S5. Analysis of concentrations of Na^+ and K^+ and K^+/Na^+ratio in *35S::BnRH6* (*AtOE*s) root and shoot. Four-week-old WT and *35S::BnRH6* plants treated with 200 mM NaCl or without for 4 d (A, B) Na^+ concentration of root and shoot in WT and *35S::BnRH6* lines. (C,D) K^+ concentration of root and shoot in WT and *35S::BnRH6* lines. (E-G) WT and *35S::BnRH6* K^+/ Na^+ ratio of root and shoot in WT and *35S::BnRH6* lines. The root and shoot were harvested, and analyzed the concentration of Na and K using ICP-AES. Vertical bars represent mean values ± SD. Different letters indicate significant differences between WT and AtOE plants ($P < 0.05$).

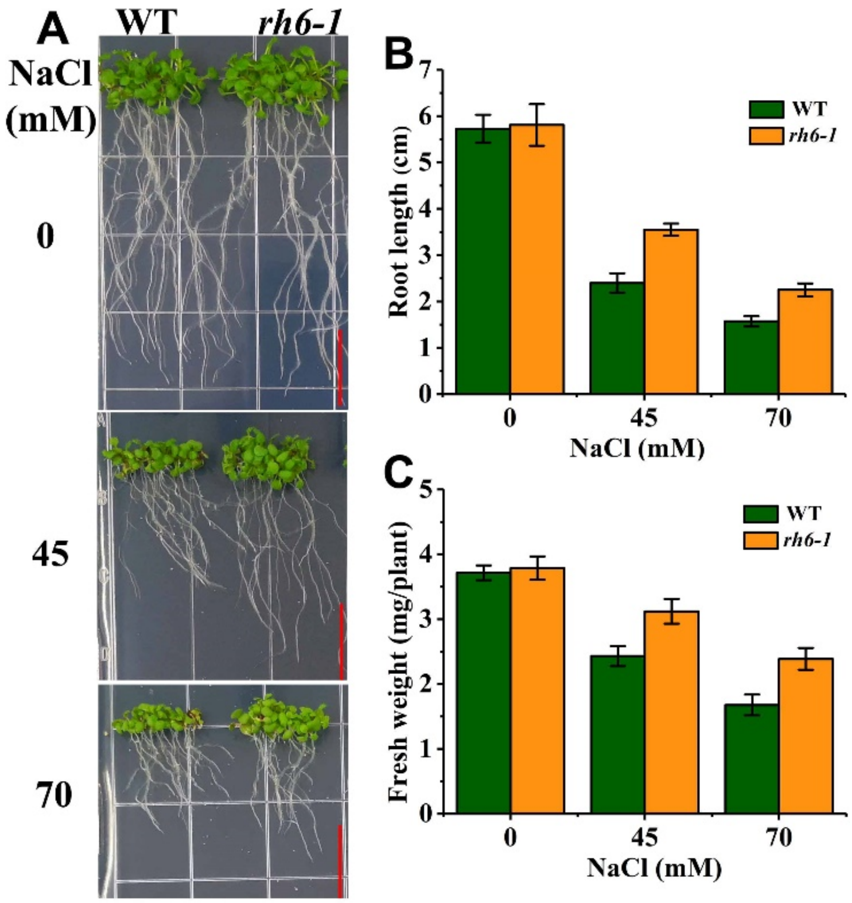

Supplementary Figure S6 Elongation of primary root and plant growth of *rh6-1* mutant under salt stress. (A): Phenotypes of *rh6-1*. (B, C): Root length and fresh weight. WT and *rh6-1* seeds were grown in 0, 45, and 70 mM NaCl 1/2 MS medium for 14 d. The root length and fresh weight were measured. Vertical bars represent mean values ± SD. Scale bar=1.5 cm.

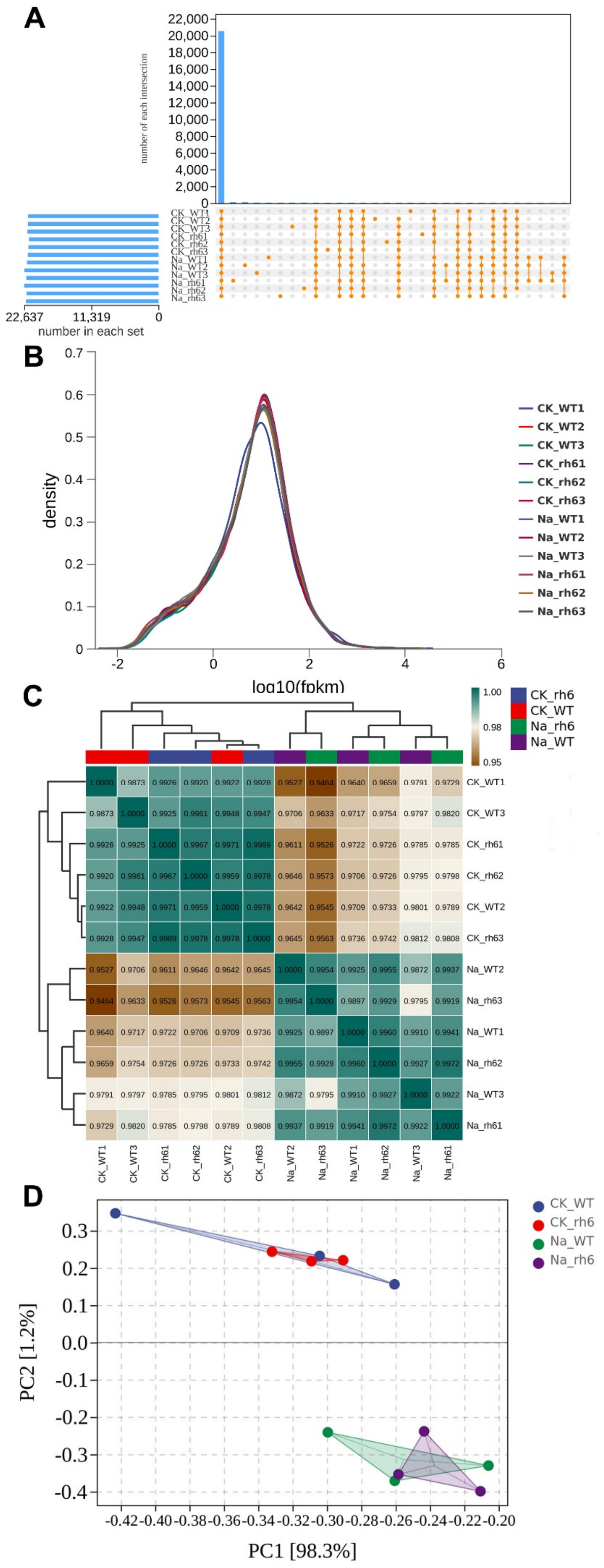
A
number of each intersection
number in each set
22,637
11,319
0
CK_WT1
CK_WT2
CK_WT3
CK_rh61
CK_rh62
CK_rh63
Na_WT1
Na_WT2
Na_WT3
Na_rh61
Na_rh62
Na_rh63
B
density
log10(fpkm)
CK_WT1
CK_WT2
CK_WT3
CK_rh61
CK_rh62
CK_rh63
Na_WT1
Na_WT2
Na_WT3
Na_rh61
Na_rh62
Na_rh63
C
CK_rh6
CK_WT
Na_rh6
Na_WT
D
PC2 [1.2%]
PC1 [98.3%]
CK_WT
CK_rh6
Na_WT
Na_rh6

Supplementary Figure S7. Analysis of the RNA-seq quality regarding the expression between the datasets. (A) Overall number and distribution of expressed genes; (B) FPKM density map of expressed genes, (C) correlation of gene expression levels among 12 samples. (D) PCA analysis. FPKM: Fragments Per Kilobase of transcript per Million mapped reads. PCA: Principal Component Analysis.

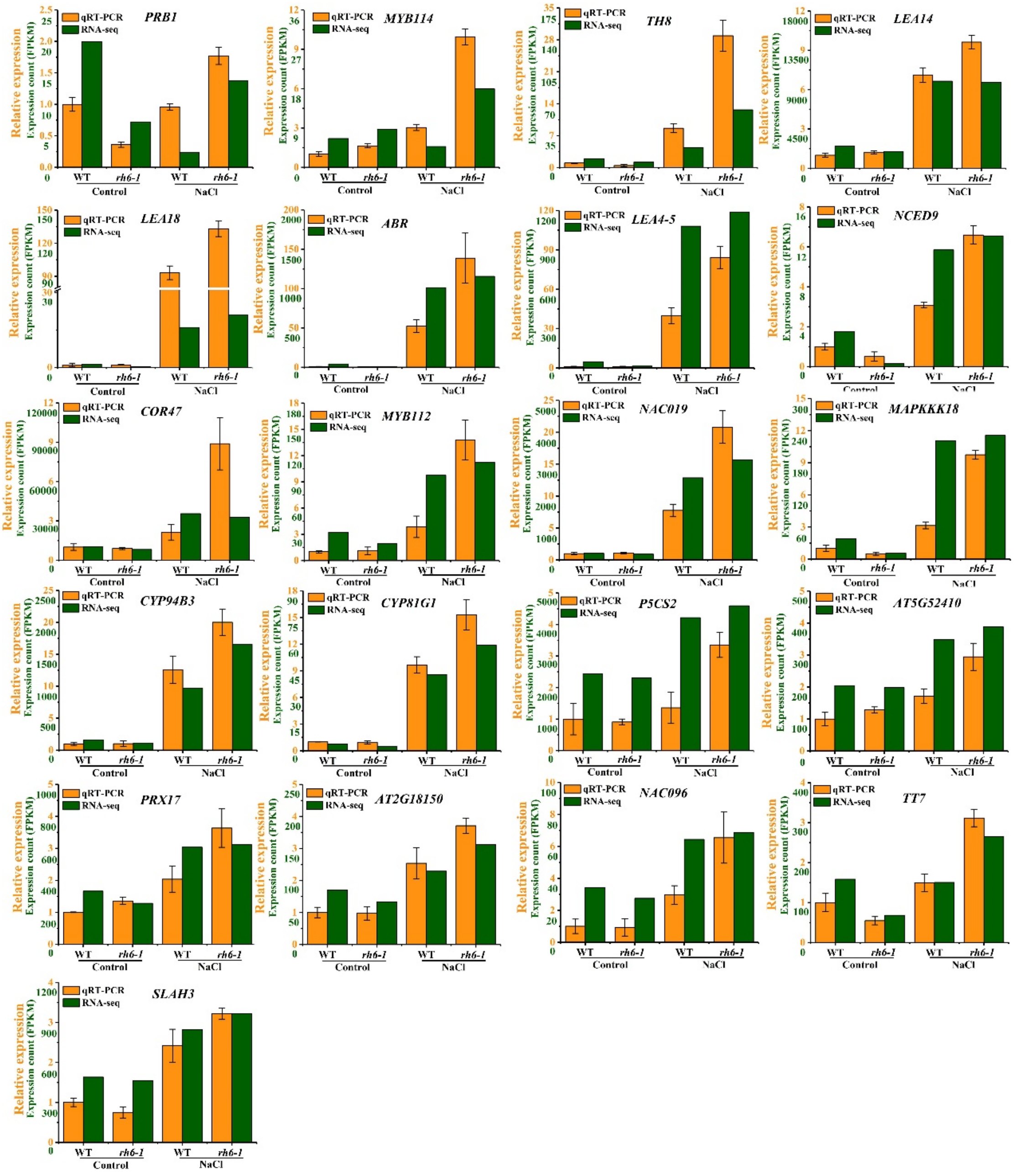

Supplementary Figure S8. Quantitative RT-PCR validation of RNA-seq datasets. The plant materials used for RT-PCR were the same as being used for RNA-seq (see Materials and methods). Vertical bars represent mean values ± SD (n = 3).

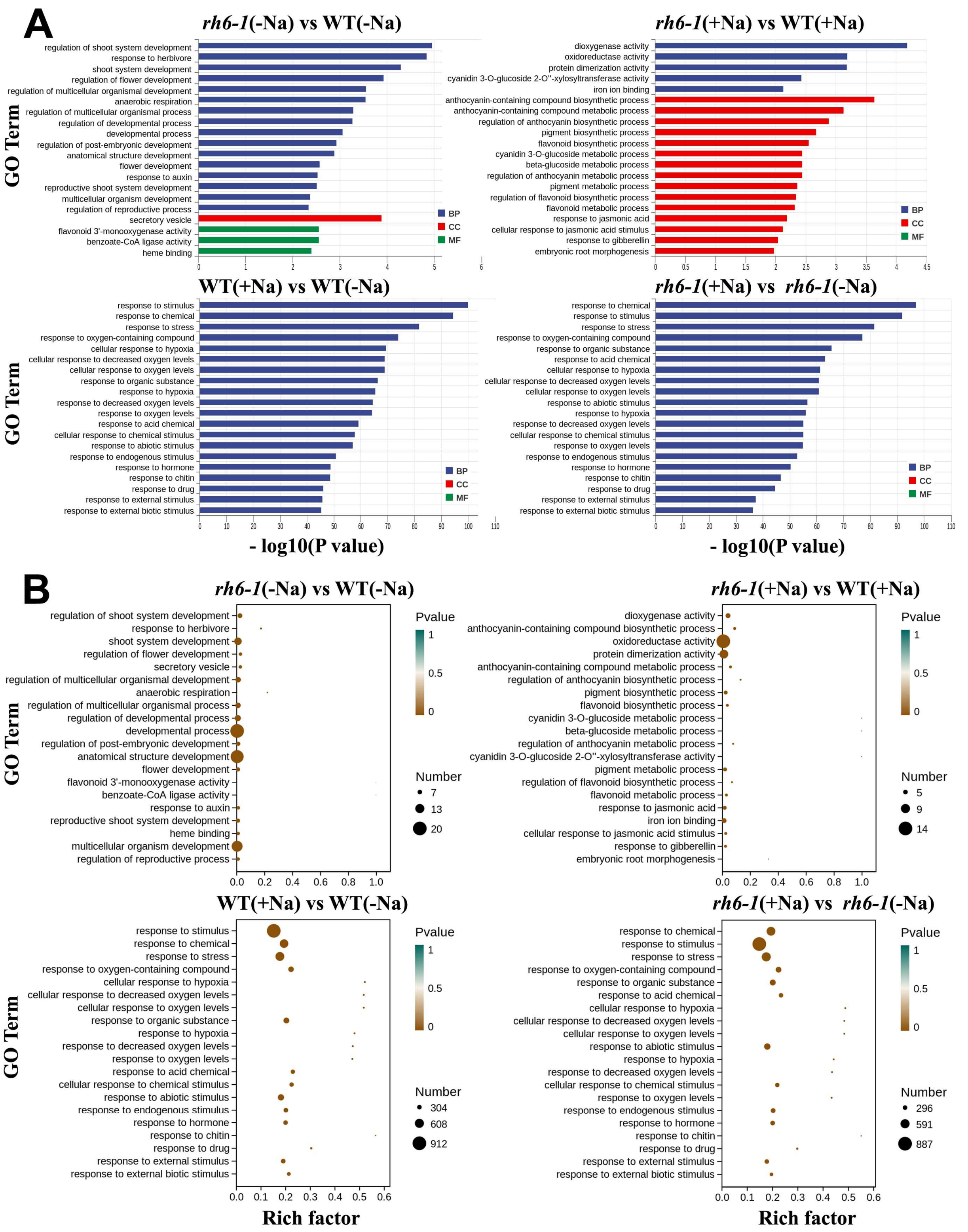

Supplementary Figure S9. GO enrichment analysis of the RNA-seq data. (A): Histogram: The abscissa is GO Term, and the ordinate is -log10 (p-value) enriched by GO Term (B): Factor graph: the abscissa is rich factor (the number of differential genes annotated to GO Term/total number of genes annotated to the GO Term), the ordinate is GO Term, and the size of the dot in the figure represents the difference annotated in the corresponding term (Up-regulation or down-regulation is related to the gene set selected in the analysis). The number of genes and the shade of the color indicate the level of significance. GO: Gene Ontology.

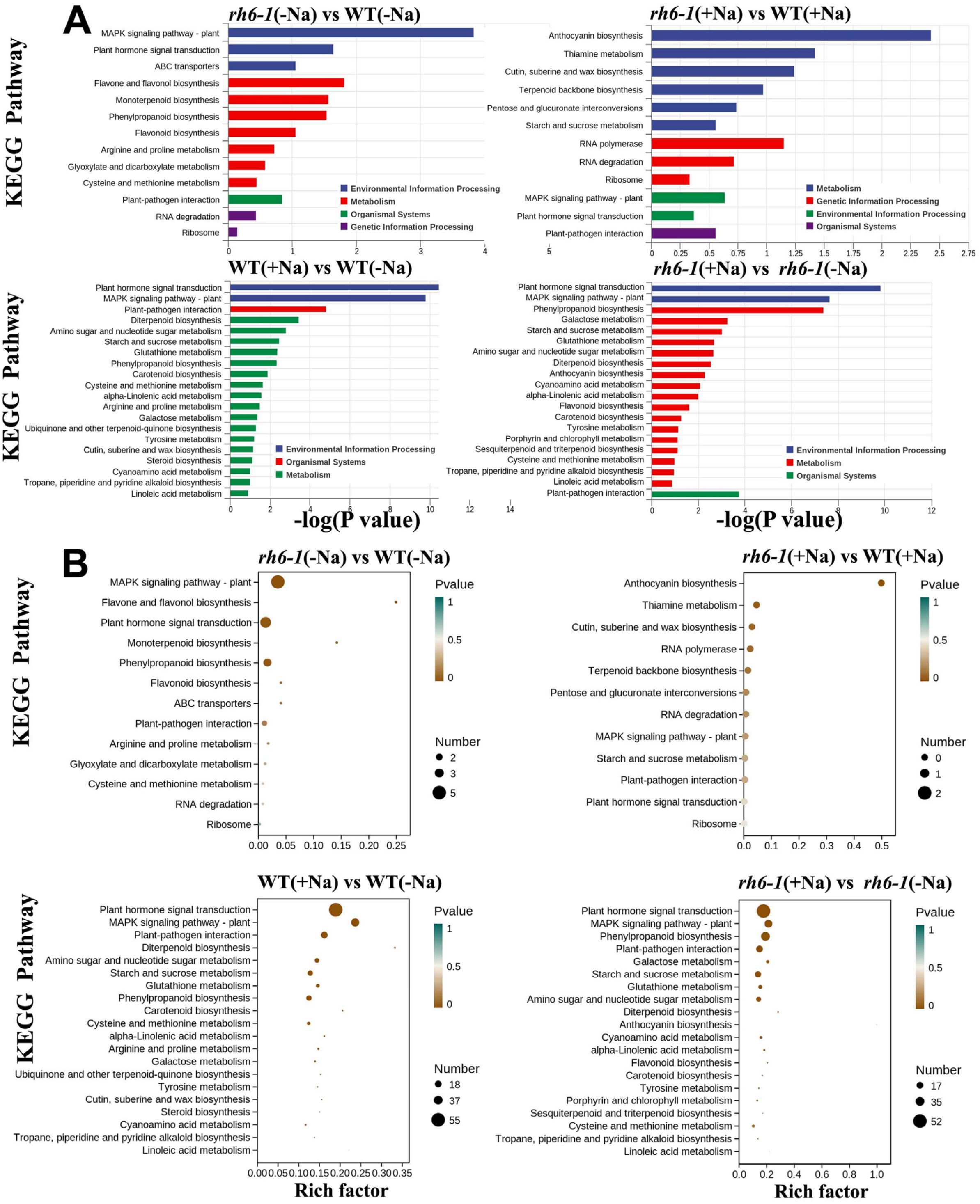

Supplementary Figure S10. KEGG enrichment analysis of RNA-seq data. (A) Histogram: The abscissa is the pathway, and the ordinate is -log10 (p-value) enriched by the pathway. (B) Factor graph: the abscissa is rich factor (the number of differential genes annotated to the pathway/total number of genes annotated to the pathway). The ordinate is the pathway, and the size of the dot in the figure represents the difference annotated in the corresponding pathway (up or down). It is related to the gene set selected in the analysis. The number of genes and the shade of the color indicate the level of significance. KEGG: Kyoto Encyclopedia of Genes and Genomes.
