# Supplementary material for: Identification of a DEAD-box RNA Helicase BnRH6 Reveals Its Involvement in Salt Stress Response in Rapeseed (Brassica napus)
Source: Int J Mol Sci. 2022 Dec 20;24(1):2. doi: 10.3390/ijms24010002 (PMC9819673; doi:10.3390/ijms24010002)
Supplement: Supplementary file 1 [file ijms-24-00002-s001.zip › Supplementary Tables.pdf]

Supplementary Tables

Table S1. Statistic analysis of raw datasets of 12 transcriptomes sequenced by RNA-seq

Sample	Raw_Read _Number	Raw _Bases	Raw_Q30 _number	Raw_N _rate	Raw_ Q20_rate	Raw_Q30 _rate
CK_WT1	45549710	6832456500	6432093108	0.001226	97.86	94.14
CK_WT2	43883614	6582542100	6177301625	0.001223	97.77	93.84
CK_WT3	47141280	7071192000	6626847880	0.001226	97.78	93.71
CK_rh61	42137674	6320651100	5935770747	0.001234	97.85	93.91
CK_rh62	42322918	6348437700	5971155629	0.001224	97.89	94.05
CK_rh63	46979196	7046879400	6612994377	0.001226	97.81	93.84
Na_WT1	39834784	5975217600	5621831084	0.001219	97.95	94.08
Na_WT2	46022274	6903341100	6488722886	0.001227	97.9	93.99
Na_WT3	39096120	5864418000	5521985185	0.001226	97.9	94.16
Na_rh61	48641282	7296192300	6866373820	0.001244	97.94	94.1
Na_rh62	44962660	6744399000	6349205996	0.001231	97.94	94.14
Na_rh63	43398020	6509703000	6138679821	0.001237	98.04	94.3

Sample: sample name; Reads No.: total number of reads; Bases(bp): total number of bases; Q30 (bp): total number of bases with a base recognition accuracy rate of over 99.9%; N(%): the proportion of ambiguous bases Percentage; Q20(%): The percentage of bases with a base calling accuracy rate of over 99%; Q30(%): The percentage of bases

with a base calling accuracy rate of over 99.9%.

Table S2. Sequencing and comparison results and statistic analysis

Sample	Clean _Reads	Total_ Mapped	Multiple_Mapped	Uniquely_Mapped	Map_ Events	Mapped_ to_Gene	Mapped_to _InterGene	Mapped_ to_Exon
CK_WT1	40311526	38743727 (96.11%)	915053 (2.36%)	37828674 (97.64%)	37828674	37733032 (99.75%)	95642 (0.25%)	37682248 (99.87%)
CK_WT2	39649788	38208222 (96.36%)	822126 (2.15%)	37386096 (97.85%)	37386096	37270351 (99.69%)	115745 (0.31%)	37184536 (99.77%)
CK_WT3	42715972	40960498 (95.89%)	882556 (2.15%)	40077942 (97.85%)	40077942	39945181 (99.67%)	132761 (0.33%)	39859397 (99.79%)
CK_rh61	37279904	35866770 (96.21%)	766415 (2.14%)	35100355 (97.86%)	35100355	34997552 (99.71%)	102803 (0.29%)	34925180 (99.79%)
CK_rh62	33657312	32321906 (96.03%)	668346 (2.07%)	31653560 (97.93%)	31653560	31552433 (99.68%)	101127 (0.32%)	31478242 (99.76%)
CK_rh63	41614528	40018326 (96.16%)	815778 (2.04%)	39202548 (97.96%)	39202548	39081347 (99.69%)	121201 (0.31%)	38993042 (99.77%)
Na_WT1	35459606	34205745 (96.46%)	633495 (1.85%)	33572250 (98.15%)	33572250	33463730 (99.68%)	108520 (0.32%)	33383226 (99.76%)
Na_WT2	39636476	38144635 (96.24%)	734728 (1.93%)	37409907 (98.07%)	37409907	37269708 (99.63%)	140199 (0.37%)	37176325 (99.75%)

Na_WT3	34352652	33026492 (96.14%)	673989 (2.04%)	32352503 (97.96%)	32352503	32256828 (99.70%)	95675 (0.30%)	32192818 (99.80%)
Na_rh61	43229460	41674117 (96.40%)	820089 (1.97%)	40854028 (98.03%)	40854028	40706812 (99.64%)	147216 (0.36%)	40603961 (99.75%)
Na_rh62	40770872	39226931 (96.21%)	767975 (1.96%)	38458956 (98.04%)	38458956	38333259 (99.67%)	125697 (0.33%)	38241284 (99.76%)
Na_rh63	38208210	36893282 (96.56%)	710881 (1.93%)	36182401 (98.07%)	36182401	36062412 (99.67%)	119989 (0.33%)	35982194 (99.78%)

Table S3. Number of differential exressed genes (DEGs)

Control_vs_Treat	Up-regulated	Down-regulated	Total
rh6-1(-Na) vs. WT(-Na)	28	48	76
rh6-1(+Na) vs. WT(+Na)	41	66	107
WT(+Na) vs. WT(-Na)	1840	426	2266
rh6-1(+Na) vs. *rh6-1*(-Na)	1749	494	2243

Control: Control group samples Treat: Experimental group samples Up-regulated: The number of up-regulated genes in Treat compared to Control Down-regulated: The number of down-regulated genes in Treat compared with Control Total: The total number of differentially expressed genes in Treat compared to Control .

Table S4. Screening data of 38 differentially expressed genes co-expressed in WT(+Na) vs. WT(-Na) group and *rh6-1*(+Na) vs. *rh6-1*(-Na) group

ID	Name	Description	log2FC WT(+Na) vs. WT(-Na)	log2FC rh6-1(+Na) vs. rh6-1(-Na)
AT1G01470	LEA14	Late embryogenesis abundant protein	1.996512554	2.4036186719062
AT1G52690	LEA7	Late embryogenesis abundant protein (LEA) family protein	4.683274975	7.76203774055928
AT2G18340	-	late embryogenesis abundant domain-containing protein / LEA domain-containing protein	2.878265407	4.45252149029211
AT2G35300	LEA18	Late embryogenesis abundant protein, group 1 protein	3.701090177	5.89108799966001
AT3G02480	ABR	Late embryogenesis abundant protein (LEA) family protein	4.632874957	6.88591036667151
AT3G53040	-	late embryogenesis abundant protein, putative / LEA protein	2.478871301	4.70460572712832
AT5G06760	LEA4-5	Late Embryogenesis Abundant 4-5	4.539882372	6.45958620564844
AT1G78390	NCED9	nine-cis-epoxycarotenoid dioxygenase 9	1.908552612	5.26791496288333
AT4G26080	ABI1	Protein phosphatase 2C family protein	1.992974626	2.0774609413834
AT5G59220	HAI1	PP2C protein (Clade A protein phosphatases type 2C)	3.975003221	5.53477719718475
AT1G07430	HAI2	highly ABA-induced PP2C protein 2	3.771875154	5.17445100530129
AT2G29380	HAI3	highly ABA-induced PP2C protein 3	2.609050493	2.9258190457873

AT5G51990	CBF4	C-repeat-binding factor 4	4.454259282	7.70629620301452
AT1G20440	COR47	cold-regulated 47	1.805399223	2.03059282602401
AT1G43000	PLATZ	PLATZ transcription factor family protein	1.662070114	2.9843593943149
AT1G48000	MYB112	myb domain protein 112	1.620465047	2.5844074845316
AT1G52890	NAC019	NAC domain containing protein 19	3.678489912	4.17129294092222
AT1G77450	NAC032	NAC domain containing protein 32	2.751499955	3.30032625694837
AT4G17980	NAC071	NAC domain containing protein 71	2.661295412	4.89797133196416
AT1G05100	MAPKKK18	mitogen-activated protein kinase kinase kinase 18	2.688958831	4.43640212308073
AT2G29440	GSTU6	glutathione S-transferase tau 6	1.221121727	1.73843180097519
AT2G29460	GSTU4	glutathione S-transferase tau 4	2.743137444	2.88088338783804
AT1G05340	ATHCYSTM1	cysteine-rich TM module stress tolerance protein	2.290025695	3.16437344014494
AT2G19900	NADP-ME1	NADP-malic enzyme 1	3.729119857	6.22363352107382
AT4G37710	-	VQ motif-containing protein	7.557321099	9.25835926681632
AT4G24000	CSLG2	cellulose synthase like G2	5.65345453	6.81807953065709
AT4G24010	CSLG1	cellulose synthase like G1	1.206823277	1.53614901392625
AT4G19230	CYP707A1	cytochrome P450, family 707, subfamily A, polypeptide 1	2.247034446	2.70279866158607
AT3G48520	CYP94B3	cytochrome P450, family 94, subfamily B, polypeptide 3	2.588883793	3.94537695556437
AT5G67310	CYP81G1	cytochrome P450, family 81, subfamily G, polypeptide 1	3.576857144	4.65422981385784
AT3G51860	CAX3	cation exchanger 3	2.154529861	2.33707633202786
AT5G24540	BGLU31	beta glucosidase 31	3.902452621	5.19736072481282
AT5G24550	BGLU32	beta glucosidase 32	2.515283089	3.85793471141787
AT4G12520	SSP	Bifunctional inhibitor/lipid-transfer protein/seed storage 2S albumin superfamily protein	-2.814843496	-3.04670936998362

FC: Foldchange

Table S5. Screening data of unique differentially expressed genes in *rh6-1*(+Na) vs. *rh6-1*(-Na) group

ID	Name	Description	log2FC(*rh6-1*(+Na) vs. *rh6-1*(-Na))
AT1G02820	LEA3	Late embryogenesis abundant 3 (LEA3) family protein	1.022549609
AT2G46300	LEA	Late embryogenesis abundant (LEA) hydroxyproline-rich glycoprotein family	2.15325205
AT3G55610	P5CS2	delta 1-pyrroline-5-carboxylate synthase 2	1.039314286
AT1G45145	TRX H5	thioredoxin H-type 5	1.182790575
AT1G69880	TRX H8	thioredoxin H-type 8	3.465638559
AT1G03020	-	Thioredoxin superfamily protein	1.116717435
AT3G11773	-	Thioredoxin superfamily protein	3.83360066
AT5G52410	-	oxidoreductase/transition metal ion-binding protein	1.011883281
AT2G22420	PRX17	Peroxidase superfamily protein	1.26322475
AT2G18150	-	Peroxidase superfamily protein	1.323284347
AT3G02990	HSFA1E	heat shock transcription factor A1E	1.031917002
AT5G03720	HSFA3	heat shock transcription factor A3	1.018563982
AT1G31040	PLATZ	PLATZ transcription factor family protein	2.101675002
AT3G60670	PLATZ	PLATZ transcription factor family protein	1.563699878
AT3G01600	NAC044	NAC domain containing protein 44	1.596847842
AT3G44350	NAC061	NAC domain containing protein 61	1.522756292

AT5G46590	NAC096	NAC domain containing protein 96	1.364203122
AT3G01970	WRKY45	WRKY DNA-binding protein 45	1.60039928
AT1G80590	WRKY66	WRKY DNA-binding protein 66	3.728377493
AT5G23000	MYB37	myb domain protein 37	1.224189164
AT1G68320	MYB62	myb domain protein 62	2.652715592
AT1G66390	MYB90	myb domain protein 90	2.438542411
AT5G55020	MYB120	myb domain protein 120	4.384235308
AT1G66370	MYB113	myb domain protein 113	1.949181195
AT4G34400	AP2/B3	AP2/B3-like transcriptional factor family protein	1.031630402
AT2G44910	HB4	homeobox-leucine zipper protein 4	1.171388882
AT5G07990	TT7	Cytochrome P450 superfamily protein	1.999297373
AT2G45570	CYP76C2	cytochrome P450, family 76, subfamily C, polypeptide 2	3.349050119
AT5G63450	CYP94B1	cytochrome P450, family 94, subfamily B, polypeptide 1	1.653555603
AT3G44970	P450	Cytochrome P450 superfamily protein	1.280633633
AT1G24070	CSLA10	cellulose synthase-like A10	1.713447962
AT4G16590	CSLA01	cellulose synthase-like A01	2.039357446
AT2G32530	CSLB03	cellulose synthase-like B3	1.087955198
AT5G51760	AHG1	Protein phosphatase 2C family protein	2.29985054
AT5G24030	SLAH3	SLAC1 homologue 3	1.103485336
AT3G22840	ELIP1	Chlorophyll A-B binding family protein	1.066069007
AT4G14690	ELIP2	Chlorophyll A-B binding family protein	1.119678695
AT5G17220	GSTF12	glutathione S-transferase phi 12	1.257641501
AT5G58080	ARR18	response regulator 18	3.401500592
AT4G22666	SSP	Bifunctional inhibitor/lipid-transfer protein/seed storage 2S albumin superfamily protein	-2.323607147
AT5G46900	SSP	Bifunctional inhibitor/lipid-transfer protein/seed storage 2S albumin superfamily protein	-1.658086795

FC: Foldchange

Table S6. Screening data of co-expressed differentially expressed genes in *rh6-1*(-Na) vs. WT(-Na) group and *rh6-1*(+Na) vs. WT(+Na) group

ID	Name	Description	log2FC (*rh6-1*(-Na) vs. WT(-Na))	log2FC (*rh6-1*(+Na) vs. WT(+Na))
AT1G31040	ORE15	PLATZ transcription factor family protein	-1.31519	2.006199504
AT2G14580	PRB1	basic pathogenesis-related protein 1	-1.46791	5.908559883

FC: Foldchange

Table S7. Screening data of unique differentially expressed genes in *rh6-1*(+Na) vs. WT(+Na) group

ID	Name	Description	log2FC (*rh6-1*(+Na) vs. WT(+Na))
AT1G66370	MYB113	myb domain protein 113	1.090838
AT1G66380	MYB114	myb domain protein 114	1.915329
AT3G27920	MYB0	myb domain protein 0	1.379517
AT5G35770	SAP	Transducin/WD40 repeat-like superfamily protein	3.940667
AT4G31615	-	Transcriptional factor B3 family protein	1.825947
AT4G15680	GRXS4/ROXY13	Thioredoxin superfamily protein	1.041717
AT1G69880	TH8	thioredoxin H-type 8	1.534248
AT3G55970	JRG21	jasmonate-regulated gene 21	1.158753
AT5G35960	-	Protein kinase family protein	1.143613
AT5G64910	-	Serine/Threonine-kinase	1.373233
AT3G60650	-	transmembrane protein	3.123632
AT4G22666	-	Bifunctional inhibitor/lipid-transfer protein/seed storage 2S albumin superfamily protein	-1.46899

Table S8. Primer and probe sequences used for this study

Application	Gene name	Forward (5'-3')	Reverse (5'-3')
Cloning	*BnRH6*-OE	TACCACTAGTCCCGGGGGATCCA TGGATAACAATAACAAC	CCCTTGCTCACCATGTCGACTTAC TGACAGTAGATTG
Subcellular localization	*BnRH6-GFP*	CTTAAGTCCGGAGCTAGCTCT AGAAT GGATAACAATAACAAC	CCCTTGCTCACCATGGATCCTTACT GACAGTAGATTG
	AtDCP1-RFP	CTGAGTTTTTCTGATTAACAGGGATCCA TGTCTCAAAACGGGAAGATAAT	CGCCCTTGCTCACCATGGATCCTTGTT GAAGTGCATTTTGTAAAG
Mutant identification	*rh6-1* SAIL_111_h08	TCCAACCAGTAAATGGACAGG	ATAGAGGAAGATTTCCACCGG
	LB3	TAGCATCTGAATTTCATAACCAATCTCGATACAC	
qRT-PCR	*BnRH6*	GCGTTTATATCAACCTGTCCAC	GAAGTACTTCACAGTGACAGGA
	AtRH6	GGTAGGCAGATCAGGACGATT	CGATAAGTGAAGGTATGGGCTTG
	PRB1	TTGCTGCCGTGAACTTGTG	CGACCTGAGTGTAGTGACCG
	MYB114	GTGGTCCTTGATTGCTGGTC	ACACGGTTCATGCTTCTTACTC

TH8	AATCTAGGCTCAACGCTCTTAA	CGACATCAATCTTCACGAACTC
LEA14	AGGACTTCGTGGCGGATAA	GTAAGGATTGGTGACAGAGACT
LEA18	TCATGCGGAGAAGACGATGG	GCTCAGCCTTGGATTGATGGA
ABR	CGGTGGAATGATGGACAAGG	GACGACATCAGCAGCTCCT
LEA4-5	ACAGATGGCTACACAGGTTAA	TGTCCAGTGGTCGAGTGA
NCED9	AATCTTCCGTCTCGTCTTCTCT	TCAAGTTGGTGACATCGTTACA
COR47	CCAGGACACCACGACAAGA	CACCACACTCTCCGACACT
MYB112	CGTTCTGCTGGACTGAATAGA	ACGAGAATGGAGTTCAAGGATG
NAC019	CGGTCTTGCGGATACTTCTAAC	CGTCTTCAGGTAGCCACAGT
MAPKKK18	CCTTGCTCGCTTACGGAAC	GTAGTTGACTCGCTGTCCATC
CYP94B3	CGGTGGAGGCATATTCAATGTC	CTAAGCGAGCGAGTGGAGAA
CYP81G1	TTGTCCACCGCTCGTCTCA	CACTGCCTCCTGATGTTACCA
P5CS2	TCACCACGAGTACAGTTCCA	TCACTATCTTCCGTCACTATGC
AT5G52410	CTCCTTCTACAGTTCCACAAGT	GGCAGAGCATCGGATTCAAT
PRX17	CAATAGGTCAAGGTCGGTGTT	CTCCAGTCACATTCTCATCTCC
AT2G18150	AAGGCAGCATTAGAGAACGAAT	ACCAGTAAGAACAGAGGAGTC
NAC096	CCGTTAGGCGATAGAAGCAAT	CATAGCAACACGACATAACACA
TT7	ACCGATGGAGACTGTTGAGAA	TTAGCGTTCCAACCTCTTCCT
SLAH3	ACAGGTACAACTCGTTCAAGAC	CAGGCATGGCTTCATTGGTAT
AtActin2	AGTGGTCGTACAACCGGTATT	GATGGCATGAGGAAGAGAGAA
BnActin2	CTCTTTCACACGCCATCCTCC	GATTCCAGCAGCTTCCATTCC

BiFC	*BnRH6*-2YN	CCATTTACGAACGATAGTTAATT AACATGGATAACAATAAC	ACTGCCACCTCCTCCACTAGTC TGACAGTAGATTGCC
	BnDCP5-2YC	CCATTTACGAACGATAGTTAATT AAATGGCGACTGATAACACGG	ACTGCCACCTCCTCCACTAGTC ACACCTCTCCCTTGGCCA
Vector	pCAMBIA 1300	GGGATGACGCACAATCCCAC	AACTTGTGGCCGTTTACGTCG
	pCAMBIA 1305	CACTATCCTTCGCAAGACCCTTC	AGATGAACTTCAGGGTCAGCTTG
	UBQ10-mcherry	GGGTTTCATAGATATCATCCG	CTCCATGTGCACCTTGAAG
	pGBKT7	GTATCGCCGGAATTTGTAATACG	CATAAATCATAAGAAATTCGCCCG
	pGADT7	GACTCACTATAGGGCGAGCG	GTGCACGATGCACAGTTGAAG
	2YN	GCAATCAAGCATTCTACTTCTATTG	GAACTTGTGGCCGTTTACGTCG
	2YC	GCAATCAAGCATTCTACTTCTATTG	GGATCTTGAAGTTCACCTTGATG
